# Live imaging of microglia during sleeping sickness reveals early and heterogeneous inflammatory responses

**DOI:** 10.3389/fimmu.2023.1253648

**Published:** 2023-09-13

**Authors:** Nestor L. Uzcategui, Sena Güçer, Cris Richter, Annika Speidel, Elizabeta Zirdum, Michael Duszenko, Olga Garaschuk, Katherine Figarella

**Affiliations:** ^1^ Department of Neurophysiology, Institute of Physiology, University of Tuebingen, Tuebingen, Germany; ^2^ Institute of Tropical Medicine, University of Tuebingen, Tuebingen, Germany; ^3^ Institute of Anatomy, Central University of Venezuela, Caracas, Venezuela

**Keywords:** Trypanosoma brucei, microglia, meninges, inflammation, heterogeneous response, immune cell recruitment

## Abstract

**Introduction:**

Invasion of the central nervous system (CNS) is the most serious consequence of *Trypanosoma brucei* infection, which causes sleeping sickness. Recent experimental data have revealed some more insights into the disease during the meningoencephalitic stage. However, detailed cellular processes befalling the CNS during the disease are poorly understood.

**Methods:**

To further address this issue, we implanted a cranial window on the cortex of B6.129P2(Cg)-*Cx3cr1^tm1Litt^*/J mice, infected them with *Trypanosoma brucei* expressing RFP via intraperitoneal injection, and monitored microglial cells and parasites longitudinally over 30 days using *in vivo* 2-photon imaging. We correlated the observed changes with histological analyses to evaluate the recruitment of peripheral immune cells.

**Results and discussion:**

We uncovered an early involvement of microglia that precedes invasion of the CNS by the parasite. We accomplished a detailed characterization of the progressive sequence of events that correlates with microglial morphological changes and microgliosis. Our findings unveiled a heterogeneous microglial response in places of initial homeostatic disruption near brain barriers and pointed out an exceptional capability of microglia to hamper parasite proliferation inside the brain. We also found early signs of inflammation in the meninges, which synchronize with the microglial response. Moreover, we observed a massive infiltration of peripheral immune cells into the parenchyma as a signature in the final disease stage. Overall, our study provides new insights into the host-pathogen immune interactions in the meningeal and parenchymal compartments of the neocortex.

## 1 Introduction

The disease caused by the protozoal extracellular parasite *Trypanosoma brucei*, known as sleeping sickness or Human African trypanosomiasis (HAT), has a devastating impact on human health, especially in the Democratic Republic of the Congo and Central Africa (1). After infection, there is a progressive invasion of the blood and lymphatic systems (disease early stage, also known as hemolymphatic stage), and later, of the central nervous system (CNS) (meningo-encephalitic stage or disease late stage). In the late stage of HAT, when the disease becomes barely treatable, clinical manifestations include severe sleep disturbances, neurological and psychiatric disorders, coma, and eventually, death without therapeutic intervention (2).

An early outgrowth of parasites in the mammalian host is restricted by the immune response of macrophages and dendritic cells, which are the primary immune cells involved in the disease (3). The host-parasite coexistence for months or even years devoid of encephalitic symptoms indicates an interplay of intricate mechanisms between the host’s immune response and the parasite’s evasion strategy, which prevents an early CNS compromise.

The brain is protected from pathogens by different specialized barriers that include physical, physiological, and immune components (4). They allow preserving constant conditions of the interstitial fluid (ISF) inside the parenchyma. These barriers encompass the blood-brain barrier (BBB) and the epithelial barrier. The BBB separates the blood from the brain. It is composed of the vascular endothelium, which fine-tunes a bidirectional flow of ISF (4), and other cells like pericytes, astrocytes, and microglia that maintain the barrier integrity and regulate the transport of nutrients and waste products between the blood and the brain (5). The epithelial barrier separates CSF from the brain parenchyma. It comprises the pia/arachnoid brain barrier, the ependyma lining the ventricle wall, and the choroid plexus responsible for CSF production and serves as a barrier separating the blood from the CSF (4). In contrast to these impermeable barriers some specialized structures in the brain collectively called the circumventricular organs (CVO) are surrounded by highly permeable capillaries that allow for the exchange of substances between the blood and the brain but also make these areas vulnerable to systemic pathogen invasion. Different subsets of immune cells present in the choroid plexus and meninges, or microglia in the parenchyma are responsible for the defense against pathogens that overcome physical barriers (5).

Microglia are highly dynamic cells (6, 7) and possess a set of receptors (microglial sensome) (8) that allows them to efficiently identify changes in the CNS microenvironment. Operating as a network of vigilant resident immune macrophages, microglia are capable of sensing pathogen-, disease-, and neurodegeneration-associated molecular patterns (PAMPs, DAMPs, and NAMPs) (9–11). The microglial sensome consists of around 100 genes (8, 12), which enable these cells to combat invading microorganisms, clear potentially harmful debris, foster tissue repair by releasing growth factors, and ultimately facilitate the restoration of tissue balance. During development and homeostasis, microglia carry out a range of non-immune tasks that are crucial for brain function, including synaptogenesis and synaptic plasticity (13–17). Under this specialized status, microglia possess a highly ramified morphology and are called homeostatic microglia (18, 19).

Despite the predictable involvement of microglia in the disease caused by *Trypanosoma brucei*, studies demonstrating their role in the control and/or progression of HAT have been scarce hitherto. *Post-mortem* analysis of brain tissue from patients who died of HAT revealed diffuse microglial hyperplasia and formation of microglial nodules (20, 21). In murine models of HAT, histological features of glial cell activation have also been reported (22–24). Studies performed *in vitro* to decipher interactions between microglia and parasites have shown that the parasite or parasite-derived molecules promotes microglial responses (25–27). Earlier, we demonstrated that freshly isolated microglia from C57BL/6 mice responded to the presence of bloodstream forms of *T. brucei*, increasing nitric oxide production and engulfing parasites (26). The impact of this phenomenon on the parasite’s elimination *in vivo* seems to be critical but remains unknown.

Here we develop an experimental model to study long-term *in vivo* imaging of individual microglia during Trypanosoma brucei infection. With this model, we tracked the morphological changes of individual microglia cells, longitudinally within the mouse’s brain parenchyma in real-time, alongside the disease progression. The observations obtained were further supported by immunohistochemical studies and ultimately associated with the course of the disease. Our findings unveiled a complex, continuous, and progressive heterogeneous microglial response that initiates in the hemolymphatic stage of the disease. We anticipate that this new approach will allow researchers to further unravel, at the single-cell level, in a highly precise and timely manner, the evolution of events that define the intricate connection among pathogenesis, immune response, and even treatment efficacy in the meningoencephalitis stage of sleeping sickness.

## 2 Materials and methods

### 2.1 Experimental animals

In this study, four to six-months-old transgenic mice expressing the enhanced green fluorescent protein (EGFP) under the Cx3cr1 promoter B6.129P2(Cg)-*Cx3cr1^tm1Litt^*/J (here referred to as CX_3_CR1^GFP/+^) were employed. In CX_3_CR1^GFP/+^ mice the expression of EGFP is achieved by placement of the EGFP reporter gene into the *Cx3cr1* locus encoding the chemokine receptor CX_3_CR1, which is expressed in immune cells such as macrophages and microglia (28). In addition, a cohort of four to six-months-old wild type C57BL/6 mice was included to evaluate the responses against infection using immunohistochemistry. These wild-type mice also served as controls by comparing all infection parameters with those observed in CX_3_CR1^GFP/+^ mice. Animals were housed under specific pathogen-free conditions, a 12:12 h light: dark cycle, and *ad libitum* access to food and water at the Experimental Core Facility serving the Neurophysiology Department, University of Tuebingen. Initially, animals were group-housed in polycarbonate cages equipped with environmental enrichment tools. After surgery and during experimental infection, animals were housed individually in the same enriched environment.

Anesthesia and euthanasia: In this study, mice were anesthetized under two circumstances, namely for cranial window implantation and during imaging sessions. For the former, the 3 components narcosis (fentanyl 0.05 mg kg^-1^, midazolam 5.0 mg kg^-1^, medetomidine 0.5 mg kg^-1^) was administered through intraperitoneal injection. For the latter, isoflurane (induction: 2%, maintenance: 0.8–1%) was applied. Euthanasia was achieved by deep anesthesia through intraperitoneal injection of ketamine/xylazine (200 mg kg^-1^ ketamine/20 mg kg^-1^ xylazine) followed by transcardial perfusion with ice-cold PBS.

### 2.2 Parasite strain and infection

Bloodstream forms (BSF) of *Trypanosoma brucei brucei* strain GVR/35, expressing the red fluorescent protein mCherry (Tbb GVR/35-mCherry, kindly provided by Jeremy Mottram and Elmarie Myburgh, University of York, UK), were used to visualize the parasite by intravital imaging using two-photon microscopy. In mice, this strain causes a cyclical infection that develops for up to 35 days post inoculation (dpi) if animals are not treated (29). Infection was achieved by intraperitoneal (i.p.) injection of 3 × 10^4^ Tbb GVR/35-mCherry parasites in PBS containing 5 mM glucose. Mock animals were i.p. injected with the same amount of vehicle (PBS + 5 mM glucose).

### 2.3 Animal monitoring

Mice were daily monitored starting from day 0 (day of the injection). The first time point (basal conditions) was acquired before animals were injected either with vehicle or with the parasite suspension. To maximize consistency, injections, as well as temperature and body weight measurements, were scheduled at the same time each day. Parameters like body temperature, weight, breathing rate, vigilance, mobility, wound area (for mice with an implanted cranial window), and general condition were individually evaluated. A scoring system based on general activity and physiological parameters, as shown in **Supplementary Table S1**, was used to evaluate animal well-being and control endpoint criteria. Stress severity was scored on a scale from 0 to 6 (normal score, 0; maximum severity: 6). Animals that showed a score ≥ 6 were euthanized and excluded from the study. After each observation, points obtained in all categories were added and a decision was made about the need for a new evaluation within the next 12 hours or after 24 h (see **Supplementary Table S1**). To determine parasite cell density, blood was drawn under isoflurane anesthesia from the tail vein starting from 4 dpi using a hemocytometer.

### 2.4 C57BL/6 brain dissection and immunolabelling

Experiments performed with wild type C57BL/6 mice involved i.p. injection, daily monitoring, parasitemia quantification, euthanasia after either 10, 20, or 30 dpi, brain dissection, and preparation for IHC. This cohort was made up by 30 female, 4-6-months old C57BL/6 wild type animals, divided into 6 groups, namely, 10, 20, and 30 dpi infected and 10, 20, and 30 dpi mock (n=5 per group). Briefly, mice were anesthetized at the denoted time with ketamine/xylazine (200 mg kg^–1^ ketamine/20 mg kg^–1^ xylazine) and transcardially perfused with ice-cold PBS followed by 4% formaldehyde 0,1 M phosphate buffer (PFA) (Roti-Histofix, Roth, Germany). Brains were dissected, olfactory bulbs and cerebellum were removed, and tissue was incubated for 24 h in PFA and further 24 h in 25% sucrose in PBS. Fixed brains were embedded in Tissue Tek (Sakura), frozen on dry ice, and kept at -80°C until use. Coronal brain cryosections of 50 μm were generated around the lateral ventricle area (Start: bregma 1.10 mm, interaural: 4.90 mm; End: bregma: 0.62 mm, interaural: 4.42 mm). For immunolabeling, brain sections were washed in PBS and incubated overnight at 4°C with the following primary antibodies diluted in PBS-plus (containing 5% normal donkey serum and 1% triton): Iba-1 (1:500, goat; Wako, no. 011-27991), TMEM119 (1:200, rabbit; Abcam, no. ab209064). In the case of TMEM119 staining, an additional step for antigen retrieval (30 min at 80°C; citrate buffer pH 6.0) was necessary prior to incubation with the primary antibody. Next, sections were washed in PBS and incubated accordingly for 2 h at room temperature with the following secondary antibodies in 2% Bovine Serum Albumin (Fraction V, Serva, Germany): Alexa Fluor 488 Donkey-antirabbit-IgG for Iba-1 and TMEM119 (Invitrogen, USA, no. A21206), Alexa Fluor 594 Donkey-anti-goat-IgG for Iba-1 (Invitrogen, USA, no. A11058). Finally, sections were washed with PBS and mounted on fluorescence-free Superfrost Plus microscope slides (Langenbrick, Germany) using Vectashield Mounting Medium (Vector Laboratories, USA).

### 2.5 Imaging acquisition of immunolabeled slides

Imaging was performed using an Olympus FV300 laser scanning system with a mode-locked laser (MaiTai HP, Spectra Physics) under either 20x water immersion objective (0.85, UPlanSApo; Olympus, USA) or 40x water immersion objective (0.80, NIR ApO; Nikon, Japan). The APT-user and MaiTai software were utilized to control the laser in terms of light intensity and excitation wavelength, respectively. Laser power was adjusted individually to obtain the optimal saturation signal for each exemplar. The secondary antibodies conjugated with Alexa 488 and 594 were excited at 800 nm wavelength and the emitted signal was passed through a beam splitter (580 nm). To selectively transmit the emission light of the fluorophores, a BP510/84 filter (for the green channel, Iba-1 or TMEM119) and LP568 filter (for the red channel, Iba-1) were used. To determine cell density, presence of doubles, and soma area, fields of view (FOV) in the superficial layers of the cortex, the periventricular region, and the lateral preoptic area were imaged using the 20x objective and the following parameters: 640 × 480 resolution, 4x digital zoom, 3x Kalman filter, normal scanning speed, and step size 1 µm (26 slides in total, final depth of 25 μm). For cellular reconstruction and morphometric analysis of microglia in Imaris, FOVs from the cortex were imaged using the 40x objective and a higher resolution: 1024 x 1024 resolution, 4x digital zoom, 3x Kalman filter, normal scanning speed, and step size 1 µm (26 slides in total, final depth of 25 μm).

### 2.6 Iba-1^+^ cell density and soma area in different brain regions

Making use of the Iba-1 signal from the 640 x 480 resolution images, microglial soma was analyzed by means of the GECIquant plugin (30) in Fiji ImageJ (imagej.net/Fiji, NIH, v.1.53). Briefly, background subtraction was performed for each channel separately. Using the ROI detection module, Iba-1-positive cells (without cell processes; only soma) were detected as Regions of Interest (ROIs). Cells without full cell soma or cells on z-stack borders were excluded from the analysis. Microglial cell number was detected in the visualized z-stacks per image through “3D object counter” plugin of ImageJ. Cells located on the borders without a completed cell soma acquired inside the FOV were not counted. The total volume in µm³ analyzed per image was calculated by picture size multiplied by the depth (200.32 µm x 150.24 µm x 25 µm). Cell density was expressed in cell/µm³. Additionally, these images underwent further analysis to determine nearest neighbor using the nearest neighbor distances plugin. The distance between nearest neighbors was calculated and all values obtained per condition were plotted as violin graphs.

### 2.7 Reconstruction of cortical microglia

3D high-resolution registrations of cortical microglia were analyzed offline using Imaris software (Bitplane, v.9.6). At least 15 cells per mouse (5 mice per condition, 6 conditions, n=30 mice; at least 450 cells) were reconstructed. First, the surface tool was applied to obtain microglial soma. Segmentation was made in the region of interest (x-y-z coordinates containing the soma). Smoothing was run at 0.173 and the thresholding method was applied with background subtraction based on local contrast. Volume, sphericity, and surface area data were exported into separate Excel files and used for statistical analysis and plotting using GraphPad Software (GraphPad Prism v.8.0.2). Reconstruction of microglial processes was conducted using the filament tracer tool. Briefly, a ROI covering the whole processes was defined and a starting point was settled in the middle of the cell body. The largest diameter of an extension and the thinnest of the process were individually specified. For the former, the size of soma’s diameter was chosen whilst, for the later, the diameter size at the process end was used. From this starting point the filament was reconstructed. The calculation of seed points was performed using the functions “remove seed points around starting point” and “remove disconnected segments” (default setting 0.259 µm). The absolute intensity and local contrast were used as thresholds to remove disconnected segments and to calculate process diameter, respectively. To reduce calculation time, the image outside the ROI was not analyzed afterward. After running the algorithm, accuracy was checked and rectified manually when necessary. The correction was made based on the visual signal of the respective cell. To eliminate branches calculated within the cell body, the filament was clipped at the visual intersections with the surface object (soma) and segments inside the cell body were deleted. Filament length, branch numbers, terminal points, and the number of intersections obtained from the reconstructed filaments were exported into separate Excel files and used for statistical analysis and plotting in GraphPad Software. Last, to obtain the volume occupied/surveilled by one microglia, reconstructed processes from each cell underwent convex hull tracing. The convex hull is a Matlab-XTension from Imaris that computes the envelope around a filament and creates a new surface that completely overlaps the filament object.

### 2.8 Cranial window implantation and *in vivo* two-photon imaging

Longitudinal evaluation of CX_3_CR1^GFP/+^ transgenic mice encompassed cranial window implantation, recovery time after surgery, infection with *T. brucei* by i.p. injection, daily well-being-monitoring, parasitemia quantification, as well as *in-vivo* imaging of microglia and trypanosomes almost every second day during a maximum of 30 dpi. For *in vivo* two-photon imaging studies, a cranial window was implanted 4 weeks before infection, as previously described (31). Briefly, mice were anesthetized with 3 components narcosis (fentanyl 0.05 mg kg^-1^, midazolam 5.0 mg kg^-1^, medetomidine 0.5 mg kg^-1^) and a 3-mm cranial window drilled on the right parietal bone over the somatosensory cortex between bregma and lambda sutures. A cover glass was fixed with glue to close the aperture and further sealed with dental cement. A stainless-steel head bar was also fixed to the skull with additional dental cement. After the surgical procedure, mice were woken up by means of i.p. antidote injection (flumazenil 0.5 mg kg^-1^, atipamezole 2.5 mg kg^-1^, naloxone 1.2 mg kg^-1^) and allowed to recover for 4 weeks before imaging was initiated. For each imaging session, mice were lightly anesthetized with isoflurane (induction: 2%, maintenance: 0.8–1%), put on a heating pad to keep body temperature stable around 37°C (controlled by a rectal thermometer), and attached to a customized holder by the metal head bar. The respiratory frequency was also monitored and kept stable between 80-110 breaths per minute. Using a combination of window x-y coordinates and the blood vessel pattern, reference points were chosen to find the same brain area over multiple sessions to monitor the same microglia during the experiment. Imaging was accomplished using a two-photon laser-scanning microscope (Olympus FluoView 300, Olympus, Tokyo, Japan) coupled to a mode-locked laser operating at 690 to 1040 nm wavelength (MaiTaiHP, SpectraPhysics, MountainView, CA, USA) and equipped with a 20x water immersion objective (0.85, UPlanSApo; Olympus, USA). EGFP was excited at 900 nm, while mCherry fluorescence was acquired at the excitation wavelength of 800 nm. Multiple z-plane stack images were acquired at a 2 μm z-step, at a resolution of 0.345 × 0.345 μm^2^ per pixel X–Y with a minimum depth of 120 μm, using the autofluorescence of the dura as a guide for the setting of the upper boundary of the z-stack (0 μm depth). Time-series images for parasites visualization were obtained with a frequency of 0.56 or 1.12 Hz.

### 2.9 *In vivo* longitudinal assessment of microglia morphology and number

*In vivo* images were analyzed using Fiji ImageJ (imagej.net/Fiji, NIH, v.1.53). Firstly, 3D images were normalized across time points and z-planes to obtain the same volume over time. This was necessary because of the engrossment of the meninges observed in infected mice. Normalization implied the creation of 80 µm sub-stacks (178.76 x 178.76 x 80 µm) encompassing the volume of the day 0. Next, all microglia were identified by comparing them with the same area image on the previous day image, which allowed their follow-up individually. Thereafter, using the GECI Quant plugin, sub-stacks were background subtracted, and microglial soma was selected with the ROI detection tool adjusting the threshold accordingly. Soma size was calculated and plotted. To determine microglia number, the plugin object counter was applied. Cells located on the borders of the FOV were excluded from the analysis. Cell density was expressed as a normalized value, taken as 100% the cell density of each mouse at 0 dpi. To illustrate the development of the heterogeneous morphological phenotype, for a selection of microglia, merely the soma was reconstructed using the Imaris software (Bitplane, v.9.6) as described above. Volume and sphericity were calculated at the different time points and exported as Excel files. Graphs were plotted using GraphPad.

Due to meningitis caused by the trypanosome infection, the separation of the meninges can reach 100 µm after 20 dpi and impair imaging. Therefore, a microscope with a depth penetration capacity of around 500 µm may be required for long-term *in vivo* imaging of the parenchyma after 20 dpi.

### 2.10 Parasite load inside the meninges

The rapid movement of trypanosomes in the CSF did allow their tracking by means of 3D imaging. Therefore, to estimate approximate values for parasite burden inside the meninges, FOVs of meninges were chosen for time series recordings at a frequency of 1.12 Hz. Areas and depths with the maximum trypanosome density were selected and video recorded. For each animal, all trypanosomes that appeared in two FOVs were manually counted. Results are expressed as trypanosomes/mm^2^. The average per animal per day was plotted on a heat-map.

### 2.11 Meningeal thickness

Using the dura surface as a guide for the upper border (0 µm) of the meninges, the distance between 0 µm and the parenchyma border was measured. The parenchyma border was recognized by the appearance on the FOV of the first microglia. Images were analyzed using Fiji ImageJ. Two 3D images per mouse were quantified. The difference between the distance (µm) measured at every single experimental day and at day 0 was calculated. Results are expressed as the average per mouse per day. With the purpose of illustrating a visualization of the meningeal thickness, 3D images were processed in Imaris. The orientation of the images was horizontally rotated 90° to get the lateral view of the tissue and the distance between the upper border and the parenchyma labeled.

### 2.12 Statistical analysis

Using power analysis, the minimum sample size was determined a priori. Experimental groups were comprised of five (5) animals each. For *post hoc* analysis of microglia and infiltrate, three-time points (10 dpi, 20 dpi, and 30 dpi) were evaluated. To determine microglial cell density, three brain slides per mouse were stained. From each slide, three different fields of view were imaged per every brain region evaluated (cortex, LPA, and PVR). At least fifteen (14) cortical microglia per mouse were reconstructed for morphometric analysis. For *in vivo* longitudinal imaging of microglia, each mouse was imaged thrice per week for 4 weeks. For parenchyma image acquisition, at least 3 FOVs per mouse were recorded. For meninges measurements, two areas per mouse were assessed. To determine parasite load inside meninges, at least two time-lapsed recordings of two different FOVs were counted. Statistical analysis was performed using GraphPad Prism software v.8.0.2 (www.graphpad.com). First, normal distribution of data was evaluated with Shapiro–Wilk, D’Agostino and Pearson normality tests. Distributions were considered normal if p ≤ 0.05. For two-group comparisons with normal distribution, unpaired Student’s t-test was performed. Samples with non-normally distributed data were analyzed with the unpaired Mann-Whitney test. For all statistical tests, values of p ≥ 0.05 were defined as non-significant, and p < 0.05 were defined as significant. Unless otherwise stated, data are given as median ± minimum and maximum (whiskers). Lines plots represent the mean ± SD.

### 2.13 Ethical statement

All procedures undertaken in mice were performed in accordance with the Directive 2010/63/EU of the European Parliament and the Council of the European Union and approved by the Regional Council Office (Regierungspräsidum, Tübingen) under the project number PY17/05 to KF. Every effort was executed to minimize animal suffering and to follow the 3Rs principle, without compromising the validity of the research.

## 3 Results

### 3.1 *T. brucei* infection induces mild to moderate distress signs in C57BL/6 wild-type mice

**Figure 1:**
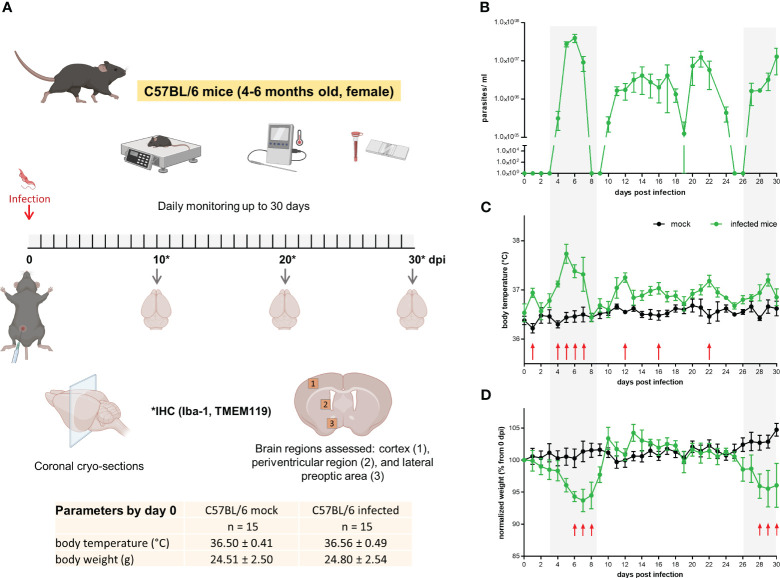
Mild to moderate distress signs in *T.brucei*-infected C57BL/6 mice. **(A)** Experimental design applied to C57BL/6 wild type mice. Basal parameters were first assessed before animals were injected with either PBS (mock) or a parasite suspension (Tbb GVR/35-mCherry) (inserted table). Every day throughout the 30 days infection, well-being was documented using a scoring system to monitor movement, vigilance, breathing rate, parasitemia, and weight. Created using BioRender. **(B)** Parasitemia course in C57BL/6 wild type mice monitored by direct counting using a hemocytometer. **(C)** Body temperature and **(D)** body weight of mock and Trypanosoma-infected mice. Body temperature was measured using a rectal thermometer. Body weight is represented as the percentage of the starting weight right before infection (n=5 mice per condition). Shadow areas in plots correspond to times where infected animals exhibited the highest stress score. Arrows represent statistically significant differences. Statistical analysis was performed by False Discovery Rate (FDR) determined using the Two-stage linear step-up procedure of Benjamini, Krieger and Yekutieli, with Q = 1%.

Tbb GVR/35 strain causes a chronic CNS pathology in mice (29); the disease develops for up to 35 days post inoculation (dpi) if animals are not treated. To correlate histopathological changes in the brain during experimental trypanosomiasis with disease progression, we established an experimental setup that included: infection of wild-type C57BL/6 mice by i.p. injection, daily well-being monitoring, parasitemia quantification, euthanasia, and tissue preparation for IHC (**Figure 1A**). Parasitemia displayed the oscillating behavior characteristic for trypanosome strains developing chronic infection (**Figure 1B**). Mock animals kept their body temperature within the physiological range (36.8 ± 0.7°C), with intra-animal variations from 0.5°C to 1°C. On the contrary, infected animals showed higher intra-animal fluctuations in the range from 1.2°C to 2.1°C throughout the 30 days of observation (**Figure 1C**). During this time, mock animals increased their body weight on average by 4.7% concerning that on day 0. Between 6-8 dpi and 28-30 dpi, infected mice showed a significant body weight loss when compared with mock mice (**Figure 1D**); on average the body weight of infected mice by the end of the experimental infection decreased by 4.5% when compared to the start values. All animals included in this study went into experiments with a normal body weight within the range for strain, age, and gender (24.7 ± 2.5 g). Clinical symptoms of the disease like piloerection, slowed mobility, or somnolence were observed alongside disease progression. The highest stress score obtained was 4 (<12h) (moderate stress) and was found in some mice either by the first parasitemia peak (5-7 dpi) or by the end of the experimental infection ≥ 26 dpi. A similar behavior was observed in infections performed in CX_3_CR1^GFP/+^ mice (**Supplementary Figure 1**). In this case, intra-animal variations in body temperature were between 0.8-1.4°C and 1.4-2.6°C in mock and infected mice, respectively. While body weight in mock animals remained fairly constant, infected animals lost an average of 9.5% from the start weight (0 dpi) by day 30. Overall, the most evident changes correlated with the first peak of parasitemia or the late meningo-encephalitic stage.

### 3.2 Microgliosis emerges right after the first parasitemia peak

**Figure 2:**
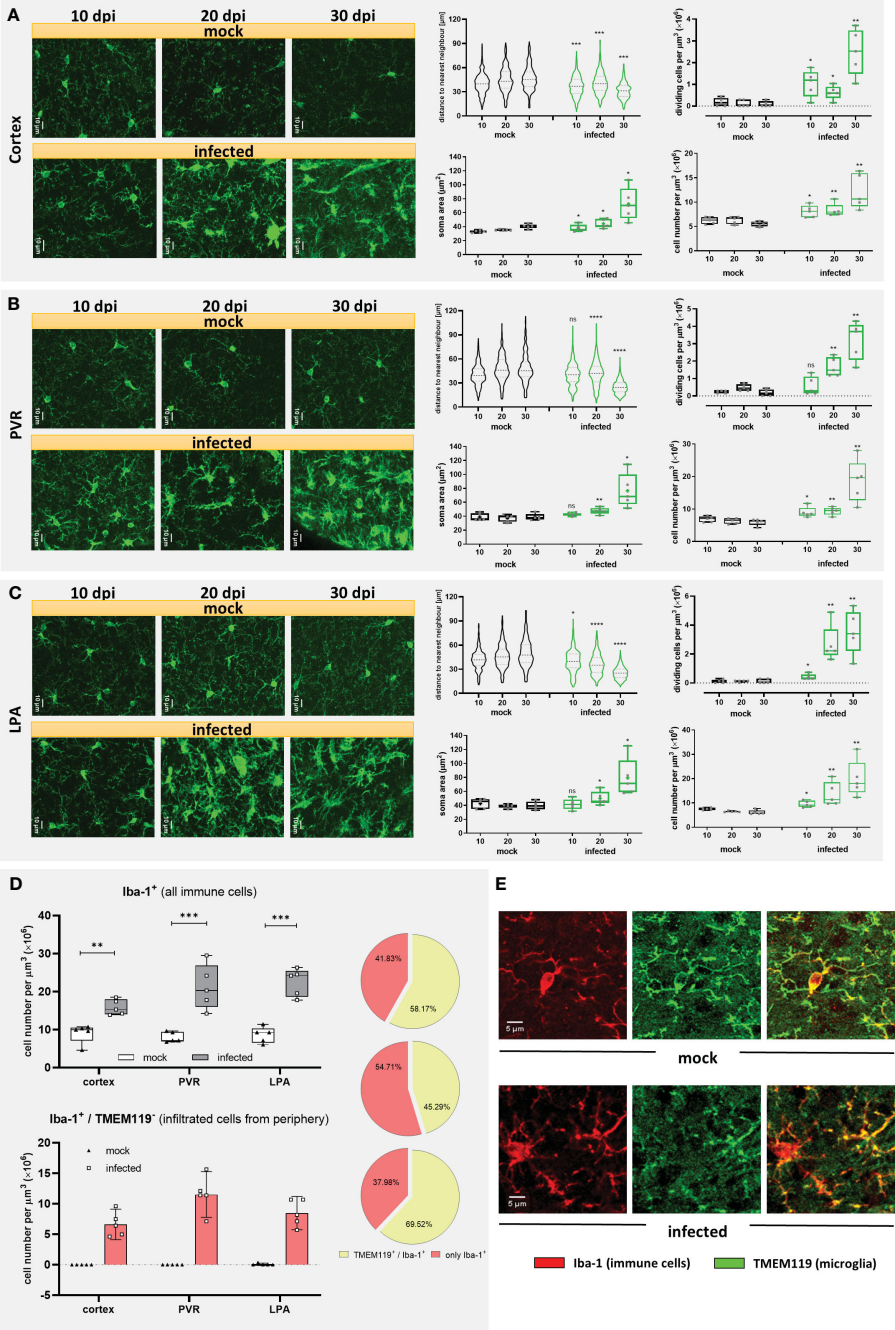
Microgliosis and subsequent infiltration of immune cells in different brain regions of *T.brucei*-infected C57BL/6 mice. After 10, 20, or 30 dpi, mice were euthanized, and brains of vehicle-treated and infected mice were used for immunohistochemical studies. Three brain areas namely the cortex **(A)**, the periventricular region (PV) **(B)**, and the lateral preoptic area (LPA) **(C)** were imaged and analyzed in mock and infected mice. On the left of each figure part, representative MIP images of Iba-1^+^ cells are shown. Violin plots show the distance to the near neighbor determined using Imaris. Whisker plots close to violin plots indicate the number of dividing cells per volume of tissue, estimated by direct counting of cells undergoing mitosis before final cell division. Whisker plots in the bottom illustrate soma area of Iba-1^+^ cells in the denoted regions (left), and Iba-1^+^ cell density (right). Data were analyzed using non-parametric Mann-Whitney test (distance to the near neighbor, dividing cells, and cell density) or parametric unpaired t-test with Welch’s correction (microglial soma area). *, p < 0.05; **, p < 0.01; ***, p < 0.001; ****, p < 0.0001; ns, non-significant. Violin plots show the median and the interquartile range of at least 85 cells. Whisker graphs display individual values per mouse (n=5 mice per condition) and represent the median (non-parametric) or mean (+, parametric) with maximum and minimum. **(D)** Recruitment of immune cells at 30 dpi in infected mice. Iba-1^+^/TMEM119^+^ double-labeled cells and Iba-1^+^/TMEM119^-^ cells were analyzed using the GECIquant plugin of ImageJ in background subtracted 3D images (25 µm depth) and manually counted. On the top, absolute number of Iba-1^+^ cells present in the cortex, PVR, and LPA at 30 dpi. On the bottom, absolute number of Iba-1^+^/TMEM119^-^ per region. On the side, pie charts showing percentage of immune cells recruited (rose-colored), and microglia (green-colored). Data were analyzed using unpaired t-test with Welch’s correction (cortex). **, p < 0.01; ***, p < 0.001. **(E)** Representative MIP images (25 µm depth, step 1 µm) showing the labeling pattern for TMEM119 in a microglial cell in a mock mouse (top) and absence of TMEM119 labeling in immune cells recruited, found in infected mice (bottom). Iba-1 signal in red. TMEM119 signal in green.

Microglia are long-lived cells that proliferate through self-renewal during the organism’s lifetime. Under physiological conditions, the microglial cell number remains relatively steady because proliferation is balanced by intrinsic apoptosis (32). To determine changes in microglia distribution, coronal cryo-slides were stained with an antibody against the ionized calcium-binding adaptor molecule 1 (Iba-1), which, in the healthy brain parenchyma, is selectively expressed in microglia (33) and is widely used in neuroscience to label microglia (34). As trypanosomiasis may affect brain regions differently, three areas were included in this analysis. Maximum projection images from the cortex (**Figure 2A**), periventricular region (PVR) (**Figure 2B**), and lateral preoptic area (LPA) (**Figure 2C**) showed significant changes in the distribution and microglial morphology in all areas. The distribution pattern of Iba-1^+^ cells, given by the distance to the nearest neighbor (dnn), showed a significantly reduced distance between cells as early as 10 dpi in the cortex and LPA (**Figures 2A, C** violin plots). In the PVR, a significant decrease in the cell-to-cell distance was found after 20 dpi (**Figure 2B**, violin plot). By the end of the experimental infection, the dnn lowered by 32.5%, 47.7%, and 47.4% in the cortex, PVR, and LPA, respectively. The fact that Iba-1^+^ cells come closer to each other is likely linked to a proliferation increase of Iba-1^+^ cells in the denoted regions in infected animals. To determine whether microglia are proliferating the number of dividing cells was assessed by direct counting of cells undergoing cytokinesis before final cell division, i.e., still sharing the cytoplasm (**Supplementary Figure 2**). An augment of dividing Iba-1^+^ cells was observed first in the cortex and LPA at 10 dpi and became most pronounced with disease progression in all studied areas (**Figures 2A-C**, upper whisker plot) thereafter. The highest numbers of dividing cells in infected mice were found in the PVR (3.20 ± 1.09 cells per µm^3^ × 10^6^) and LPA (3.53 ± 1.51 cells per µm^3^ × 10^6^) after 30 dpi. In contrast, mock animals displayed lower and steady numbers (a maximum of 0.47 ± 0.19 cells per µm^3^ × 10^6^). In line with these results, the total number of Iba-1^+^ cells increased in infected animals as early as 10 dpi in all regions analyzed in an even proportion of ~30% (**Figures 2A-C**, right whisker plots on the bottom). At 20 dpi, no major changes were found in the cortex (i.e., the observed 30% increase after 10 dpi was maintained), but in the PVR and LPA the number of Iba-1^+^ cells increased further, reaching 47% and 120%, respectively, when compared with the values of mock mice. However, a massive rise in cell numbers happened after 30 dpi, more than doubling in the cortex and more than three-fold in PVR and LPA. Beyond distribution and proliferation, an obvious enlargement of the soma size was also detected. Significant differences were found after 10 dpi in the cortex. After 20 dpi, changes were discernible in all three areas. Furthermore, at 30 dpi soma area was doubled in infected animals as compared to mock mice (**Figures 2A-C**, left whisker plots on the bottom). Taken together, the infection caused changes in microglial number and morphology after the first parasitemia wave. Alterations regarding microglial proliferation were more marked in the deeper brain regions PVR and LPA than in the cortex, but changes in soma size became earlier visible in the cortex.

### 3.3 Infiltration of immune cells follows microgliosis in infected mice

It is known that Iba-1 is not exclusively expressed in microglia but also other immune cells like peripheral monocytes and macrophages (35). As response to inflammation or infection, circulating monocytes may infiltrate the brain. To evaluate a potential migration of peripheral immune cells into the brain in our experimental infection model, a more specific marker for microglia, the transmembrane protein 119 (TMEM119) (36), was used in double staining experiments together with Iba-1. Mock mice did not show signs of infiltration, as nearly all cells displayed positive staining for Iba-1 and TMEM119. In trypanosome-infected mice, however, cells exclusively stained by Iba-1 were found only at 30 dpi (**Figure 2D**), but absent at 10 and 20 dpi. This result clearly supports microglial proliferation during trypanosomiasis but also demonstrates infiltration of peripheral immune cells towards the end of the infection. Unlike the robust Iba-1 staining, where microglial soma and processes are plainly labeled, TMEM119 staining showed immunoreactivity in processes and soma’s plasma membrane (**Figure 2E**). As judged from our immunostaining results obtained after 30 dpi, from the total amount of Iba-1 positive cells, representing microglia/macrophages, roughly 40% in the cortex and LPA and more than half in the PVR were identified as not microglia because they failed to stain with TMEM119 antibody (**Figure 2D**, pie charts). On the contrary, only a negligible number of Iba-1^+^/TMEM119^-^ cells was detected in a few mock animals (**Figure 2D**). These observations indicate a substantial increase of microglia from 10 dpi onwards in all evaluated areas of infected animals, which is strengthened by the recruitment of immune cells at the end of the infection, i.e., between 20 and 30 dpi, especially in the PVR. It is worth mentioning that although TMEM119 represents a reliable homeostatic marker for microglia, its downregulation has been reported in neurodegenerative diseases and models for demyelination (37–39). Therefore, we cannot rule out that during the late stage of the disease in our model, some microglia cells are downregulating TMEM119.

### 3.4 Infection triggers a decrease in microglial complexity

**Figure 3:**
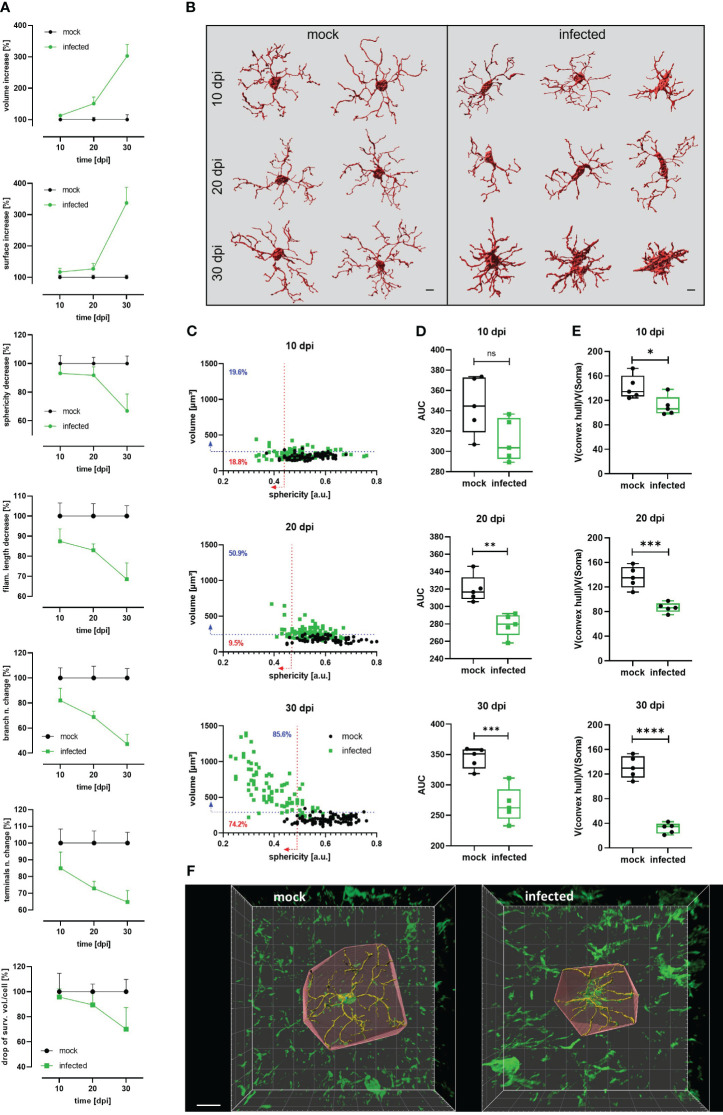
Decrease of microglial complexity in *T.brucei*-infected mice. **(A)** Morphometric analysis of cortical microglia from infected and mock C57BL/6 mice. 3D-images were acquired at high resolution (1024×1024) from 50 µm slides labeled with anti-Iba1 antibody. At least 15 microglial somas per mouse were reconstructed using the surfaces and filament tracer tools from Imaris. Lines represent the progression of each parameter normalized to time-matched values from mock animals. Each value denotes the mean plus SD of 5 animals (See **Figure S2** for absolute values of each parameter over the time). **(B)** Representative examples of reconstructed microglia from mock (left panel) and infected (right panel) mice. Morphological heterogeneity is evident at 30 dpi. Bars scale denote 5 µm. **(C)** Dot plot charts showing the distribution of soma volume vs. sphericity in mock and infected mice. Percentages on plots denote proportion of infected cells above 95^th^ percentile for volume (blue-dashed line) and below 5^th^ percentile for sphericity (red-dashed line) of time-matched mock mice. **(D)** Area under the curve (AUC) obtained plotting number of intersections relative to the distance from the microglial soma in a radius of 50 µm (step 1 µm) in mock and infected mice. **(E)** Ratio convex hull volume vs. soma volume. For each reconstructed microglia, the volume obtained for the convex hull was divided by the soma volume of the corresponding cell to obtain a V(convex hull)/V(soma) ratio per mouse. Graphs show the median with minimum and maximum. Data were analyzed using non-parametric Mann-Whitney test. *, p < 0.05; ***, p < 0.001; ****, p < 0.0001. **(F)** Representative reconstructed microglia (30 dpi) from mock (left panel) and infected (right panel) showing the convex hull. Bar scale, 10 µm.

For diverse kinds of CNS pathology, it has been described that microglia become less complex (shortening processes length and number) and, under certain circumstances, may acquire an ameboid shape (9, 40). For a comprehensive 3D quantitative morphometric analysis of microglial cells in infected and mock mice, high-resolution images of brain slices labeled by the Iba-1 antibody were acquired and analyzed using the surface and filament tracer tools of the Imaris software. Morphometric alterations of the microglial soma over time included a significant volume and surface area increase as of 10 dpi (**Figure 3A**, **Supplementary Figure 3**). Both parameters in infected animals reached more than three times the volume and surface area of mock mice at 30 dpi. Moreover, a decrease in soma sphericity was another recurrent microglial feature in infected animals (**Figure 3A**, **Supplementary Figure 3**). As shown in the **Figure 3C**, when comparing individual reconstructed cells from mock and infected mice, it becomes self-apparent that microglia’s soma in infected animals (green dots) blossoms into first less spheric (10 dpi), later turns bigger (20 dpi), and, finally, develops into an oversized soma with short and thick side extensions (30 dpi). Furthermore, microglial complexity, expressed by the filament length, the number of branches, and the filament terminals, significantly decreased in infected mice compared with microglia from mock mice after the denoted infection times (**Figure 3A**; see also **Figure 3B**). Even on 10 dpi, these parameters were reduced on average by more than 10% compared to the respective data obtained from mock animals (**Figure 3A**, **Supplementary Figure 3**). Infection also caused a decrease in the number of intersections and filament radius in microglia, represented by a smaller AUC (**Figure 3D**), which was significantly different as of 20 dpi. Microglial processes are very dynamic and are constantly monitoring the area they occupy (6, 7); therefore, the efficiency of individual microglial cells for surveying the tissue is proportional to the volume they conquer with their processes. Using the convex hull algorithm of Imaris, the surveilled tissue volume per reconstructed cell was calculated. The convex hull/soma volume ratio was plotted, it allows amending the increase of soma volume that may overestimate the factual surveilled tissue volume. A significant reduction of this parameter was found even at 10 dpi (**Figure 3E**).

### 3.5 Longitudinal *in vivo* analysis of microglia reveals early responses during the hemolymphatic stage of the disease

**Figure 4:**
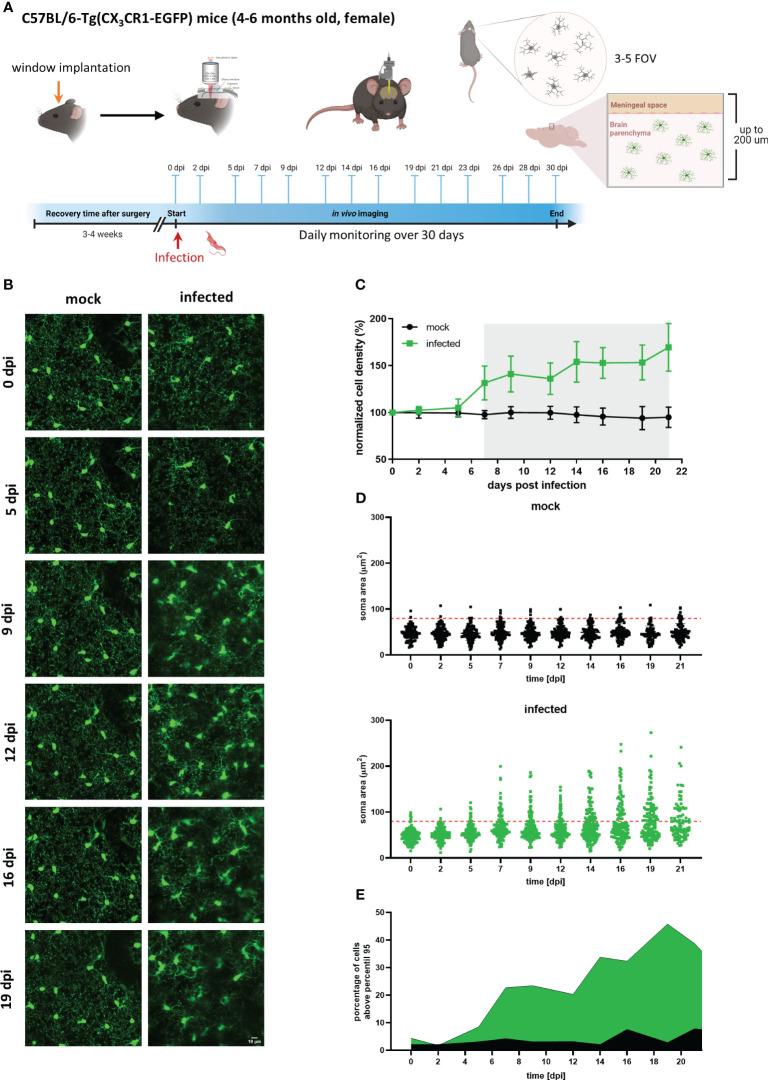
Longitudinal analysis of microglial cells in mock and infected CX_3_CR1^GFP/+^ transgenic mice. **(A)** Experimental setup to visualize by two-photon microscopy microglia in CX_3_CR1^GFP/+^ mice infected with *T. brucei* (infected) or treated with vehicle (mock). A cranial window was implanted in the somatosensory cortex. After 4 weeks recovery, mice were imaged (0 dpi) to record basal conditions before infection and then injected intraperitoneally with either a parasite suspension (3×10^4^ parasites) or with the vehicle (PBS containing 5 mM glucose). Thereafter, imaging was performed three times per week over 4 weeks. 3-5 field of views (FOV) with up to 200 µm depth were selected at 0 dpi and acquired at each imaging session over complete time. Animal well-being and parasitemia were monitored during disease progression as well. **(B)** MIP images of the same area in the cortical region showing microglial cells expressing eGFP. Images on the left are from a mock mouse (36-86 µm depth, step 2 µm). Images on the right belong to an infected mouse (38-88 µm depth; step 2 µm). Bar scale: 10 µm. **(C)** Microglial cell density in mock and infected animals. Cell numbers were obtained using the 3D object counter plugin of ImageJ in a fixed tissue volume (2.5×10^6^ µm^3^). Cell density obtained was normalized to that of day 0. Gray shadow indicates a significant difference, (unpaired t-test analysis at 7 dpi, p = 0.0018). **(D)** Microglial soma area from mock and infected mice. Soma area calculations were performed using GECI-Quant in ImageJ. Dots represent somas of individual cells, red-dashed line denote 95^th^ percentile of all analyzed microglia from mock animals. **(E)** Portion of cells categorized as non-physiological in infected mice. The percentage of cells above the 95^th^ percentile for each particular time is represented in green and black for infected and mock mice, respectively.

To characterize the timeline of events occurring during trypanosomiasis in the microglial population at the single-cell scale, CX_3_CR1^GFP/+^ transgenic mice were imaged before and regularly after infection. Mice were subjected to craniotomy for window implantation on the somatosensory cortex four weeks before they were infected with the parasite and imaged nearly every second day to visualize microglia and parasites. Our experimental setup also included daily well-being monitoring and quantification of parasites in blood (**Figure 4A**).

**Figure 5:**
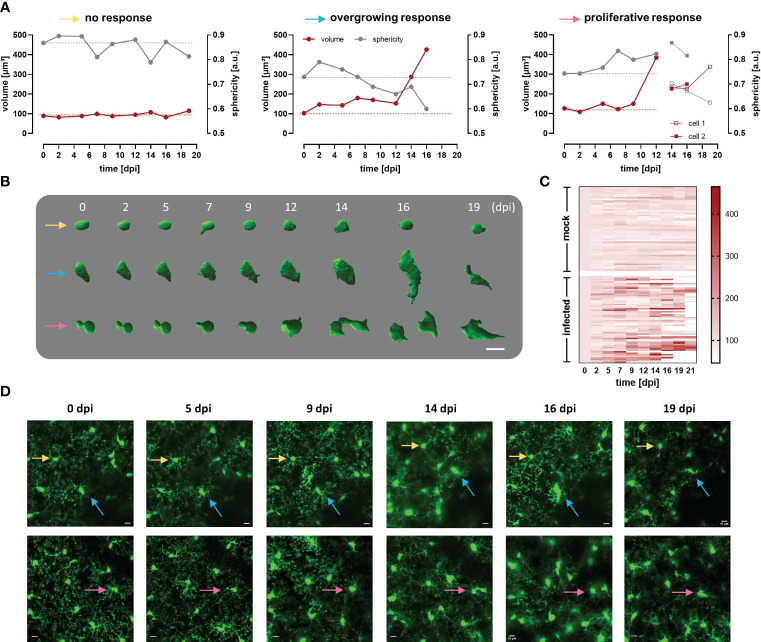
Heterogeneous response of cortical microglia in infected CX_3_CR1^GFP/+^ transgenic mice. **(A)** Changes in volume (red line) and sphericity (grey line) in three representative microglial cells that behaved differently during infection namely, cell showing non-significant changes in size and shape (yellow arrow), overgrowing cell (blue arrow), and proliferative cell (pink arrow). The last generated a daughter cell, whose volume and sphericity are also shown. Microglial volume and sphericity were obtained from reconstructed somas using Imaris Cell Imaging Software. Dotted lines indicate values at 0 dpi for volume (red) and sphericity (gray) to enable easy tracing. **(B)** Snapshots of reconstructed somas represented in A (identified with the colored arrows). Numbers denoted days post infection. Bar scale: 10 µm. **(C)** Heatmap showing volume of single microglia during a maximum of 21 days. In the upper part of the heatmap are represented microglia from 5 mock animals (n=58). In the inferior part, are displayed single microglia from 5 infected animals (n=57). Only cells that were visualized at 0 dpi but not those that appeared later in the region evaluated were considered. Cells were tracked for 21 days or until they either disappeared or migrated outside the region of interest under evaluation. Scale denotes volume (µm^3^). Microglia that are not fully represented in the heatmap till 21 dpi, are the ones that either disappeared or divided. **(D)** MIP images of two areas in the cortical region of infected mice where the reconstructed microglia were localized (see colored arrows), before parasite injection and until 19 dpi (superior panel, 42-92 µm depth; inferior panel, 18-68 µm depth; step 2 µm). Bars denote 10 µm.

Circumventing big blood vessels, three to four cortical volumes were selected at 0 dpi in each mouse. Individual microglia in those cortical volumes were longitudinally tracked three times per week until 30 dpi. Representative images of mock and infected mice acquired using λ 900 nm as excitation to stimulate GFP are shown in **Figure 4B**; mock mice displayed few changes over time such as few cell divisions and tiny migration of some cells (**Figure 4B**, left panel). In contrast, microglia in infected mice showed a highly dynamic behavior (**Figure 4B**, right panel). Up to 5 dpi, cells in the regions of interest were stable in terms of location. From 7 dpi onward, a significant increase in cell number and changes in soma shape were evident (**Figure 4B**, right panel; **Figure 4C**). To quantitatively compare microglial morphology in both the control and the experimental group, soma size was calculated using the ROI detection plugin of GECI Quant. In mock mice, soma size remained stable until 30 dpi (average_30dpi_: 48.97 ± 1.97 µm^2^). Interestingly, in infected mice, the microglial population behaved dissimilarly. Starting from 7 dpi, a wider amplitude in the soma size range was noticed, i.e., from the minimum to maximum value for a given day (mock_7dpi_: 84.13 vs. infected_7dpi_: 175.5) (**Figure 4D**). For further analysis, the 95^th^ percentile of the microglial soma size in mock animals was calculated to obtain a reliable cut-off value of what can be considered a physiological variation (doted-red line **Figure 4D**). Remarkably, the proportion of cells in infected mice that showed a size above the 95^th^ percentile from mock mice, considered to be non-physiological, indicated a bi-phasic reaction with an initial microglial response between 5-9 dpi followed by a second reboot from 12 dpi onwards (**Figure 4E**). When results were expressed not per cell, but per individuum (i.e., per mouse), significant differences in the microglial area were found as early as 5 dpi (**Supplementary Figure 4**). Furthermore, in infected mice at least three sets of microglia were distinguished: one group stayed without significant changes in size (**Figures 5A, B, D** yellow arrows), another group was characterized by enlarging cell size without cell division and the cells did typically die (**Figures 5A, B, D** blue arrows), and a third population that enlarged volume, changed its shape, and at the end generated two daughter cells (**Figure 5A, B, D** pink arrows). **Figures 5C** illustrates fluctuations in the soma area of each microglia tracked individually during 21 dpi in mock and infected mice. The heatmap exposes the heterogeneous behavior of individual microglia during the disease. Although a clear response to the infection is observed, some microglia did not change their size at all during the observed infection period, while others started to respond even 2 days post-infection. Most microglial cells begin to respond between 5-7 dpi, and although more cells become reactive during the progression of the disease, no pattern in the soma variations can be defined, apart from those described in **Figure 5B**. It is important to mention that likely due to inflammation, imaging quality in infected mice decreased with disease progression and turned a reliable quantification of morphological parameters after 21 dpi unviable. By contrast, imaging of mock mice went straightforward through the established experimental time of 30 days.

### 3.6 Parasite’s arrival into the CNS intensifies microglial response

**Figure 6:**
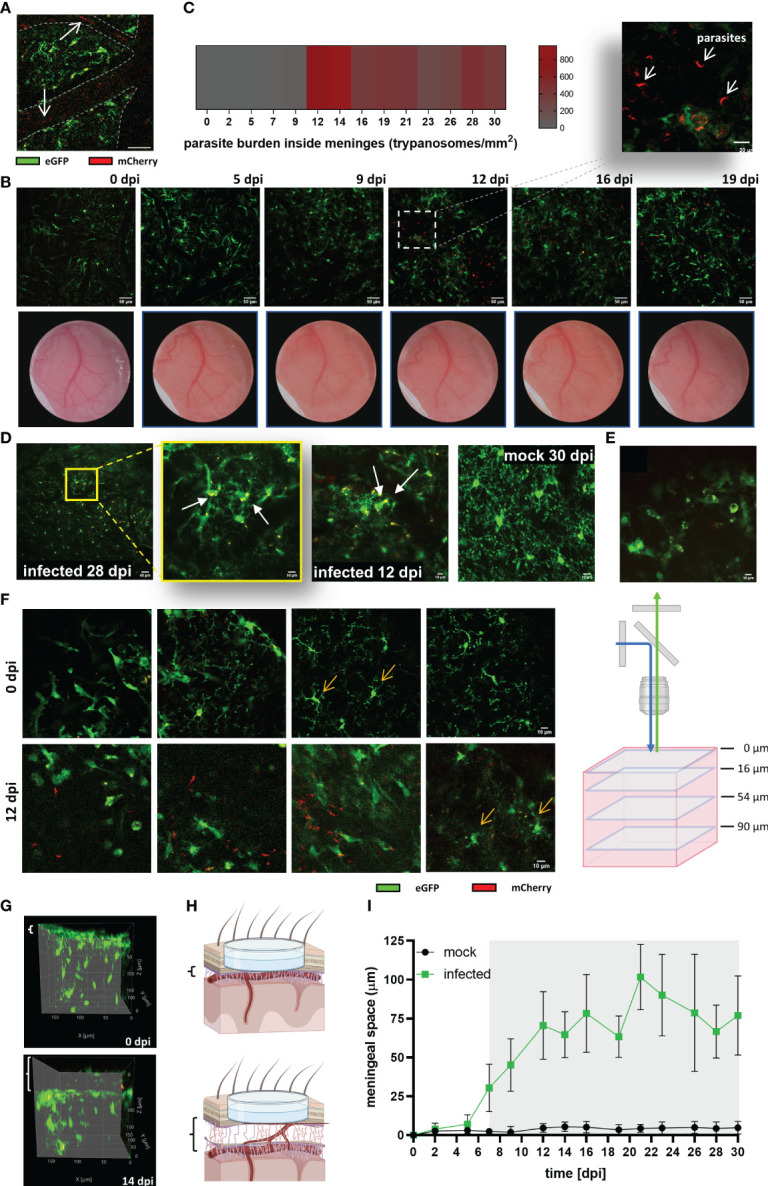
Parasite invasion and increase of the thickness of the meningeal space in infected CX_3_CR1^GFP/+^ transgenic mice. **(A)** Intravascular parasites (arrows) were detected at 5 dpi in infected mice. MIP of a cortical plane located 12 µm below the parenchyma border. The projection was made from a time series acquired at a frequency of 0.452 s; three frames are represented (40x magnification, 256×256 resolution). Blood vessels are delineated with dashed lines. Scale bar: 20 µm. **(B)** Upper panel: Extravascular parasites in the meningeal space expressing mCherry were observed at 12 dpi. MIP images showing the meningeal space close to the parenchyma border (thickness 4 µm; scale bar 50 µm). The acquisition was performed longitudinally for 19 days. Dotted square in the image 12 dpi projects an amplified image to exhibit parasite morphology (scale bar: 20 µm). Lower panel: Changes in the blood vessels pattern were visible throughout the implanted window. Clouding of the window as of 9 dpi indicative for development of oedema was noted. **(C)** Estimated parasite burden inside the meninges. Extravascular parasites swimming in a z-plane close to the pia mater were counted from time series acquired at 1.12s frequency (n = 5 mice). **(D)** Images suggestive for microglial phagocytic activity. The yellow fluorescent signal represents a merge which derives from GFP (microglia) and mCherry (expressed by trypanosomes). mCherry fluorescence was found intracellular or in the microglial processes. MIP displayed, from left to right, as follow: infected 28 dpi, lower magnification (20x, Zoom1), depth 22-34 µm; scale bar 40 µm. Amplification of the first image (20x, Zoom4) showing red cytoplasmatic material, depth 26-40 µm. Infected 12 dpi, (20x, Zoom3), showing red material in the processes, depth 26-76 µm. Mock 30 dpi (20x, Zoom4), typical image demonstrating clean processes and cytoplasm in mock animals, depth 44-80 µm. Scale bars 10 µm unless otherwise indicated. **(E)** CX_3_CR1-positive cells inside the meninges. Average intensity image from a time series acquired at 8 µm above the parenchyma border. Frequency: 1.12s per frame, 43 frames. Scale bar 10 µm. **(F)** Cortical area of an infected mouse at 0 dpi and the same area at 12 dpi (40x, Zoom4, Kalman filter 4). Single z-frames images from 0 dpi and 12 dpi are displayed to show displacement in the vertical (z-direction) of the view. On the right a schematic representation of the different frames showed. Scale bar: 10 µm. **(G)** Lateral view of the same tissue volume of an infected mouse imaged at 0 dpi, and 14 dpi. Meningeal space engrossment is indicated by the white brackets on the left of each image (8 µm at 0 dpi, and 58 µm at 14 dpi). **(H)** Schematic representation of the observed increase in the meningeal space. Created using BioRender. (**I)** Changes over the time of the meningeal space thickness in Trypanosoma-infected and mock mice. Variations with respect to the distance at basal conditions (0 dpi) were measured using ImageJ. The basal distance was subtracted from the distance measured at each experimental day. Graph display the mean ± SD from 5 mice in each condition. Gray shadow denotes statistical significance. Data were analyzed using the multiple unpaired t-test and discovery determined using the Two-stage linear step-up procedure of Benjamini, Krieger and Yekutieli, with Q = 1%. (Significance at 7 dpi, p = 0.003669).

To evaluate the parasite’s onset in the CNS, and correlate observed changes in microglia with parasite location, imaging was also performed at λ 800 nm to excite the mCherry protein constitutively expressed in trypanosomes. As expected, parasites were first detected inside blood vessels as red flash signals swimming with the bloodstream at 5 dpi (**Figure 6A**). Parasite burden in the blood (as detected by direct counting in blood samples) showed a direct relationship with lightning signals frequency. Surprisingly, the first wave of parasites in blood positively correlated with the first signs of microglial response to infection like migration, soma size increase, and later, proliferation (**Figure 4**). Extravascular parasites were visible inside the meninges usually around 12 dpi (**Figure 6B**), in few cases, they showed up as early as 7 dpi. These results are in accordance with an early study using the parasite strain GVR35 and another transgenic mouse with similar genetic background (41). By this time (12 dpi), a reboot of the microglial response was obvious as evidenced by a further increase of soma size and proliferation, which were stable after the first reaction (5-7 dpi) (**Figure 4**). To estimate (semi-quantify) the parasite load inside the meninges and because trypanosomes move extremely fast, high-frequency time-lapse recordings were acquired and parasites in the field of view were manually counted (**Figure 6C**). The highest parasite load was observed between 12-14 dpi. After this time, in all mice evaluated in this study, the trypanosome burden inside the meninges decreased but never disappeared. The maximum density of trypanosomes was found in the area just above the pia mater. However, parasites were also seen swimming freely in the CSF when imaging was displaced in the z-axis (**Suppl. Video 1**). As judged by their active movement and their capacity to proliferate in the CSF (**Suppl. Video 2**), it seems that the meninges are at least in part a permissive environment for the parasite. On the contrary, the brain parenchyma appears to be extremely restrictive to parasite permanency. Despite an intensive search for parasites inside the parenchyma, we did not observe free-moving trypanosomes. Remarkably, microglia containing red signal inside their cytoplasm (indicative for the mCherry protein) were often found in areas where morphological features of microglial response to infection were obvious (**Figure 6D**). In addition, another remarked feature during disease progression was the increase of CX_3_CR1-positive cells inside the meninges (**Figure 6E**, **Suppl. Video 3**). Diapedesis of CX_3_CR1-positive cells was a frequent phenomenon observed around 14 dpi and onwards (**Suppl. Video 4**).

### 3.7 Signs of meningitis correlate with initial microglial changes and precede parasite entry in this compartment

Inflammation of the meninges develops in response to the presence of pathogenic microorganisms that target the CNS, but also in response to several systemic diseases (42). To unveil the timepoint by which signs of meningitis appear, and uncover possible associations with the observed microglial response, the space between the upper part of the meninges and the brain parenchyma was measured in mock and infected mice. A clear indication of inflammation was a patent increase of window cloudiness in infected animals starting from 9 dpi (**Figure 6B**). Likewise, the displacement of the field of view in the z direction from the initial 0 µm plane at day 0 (zero_0dpi_), which was settled focusing the dura mater, was another evidence that the meningeal space was progressively swelling. **Figure 6F** illustrates typical images of infected mice; four different cortical depths of a unique field of view before (0 dpi, upper panel) and after infection (12 dpi, inferior panel) are shown. In this case, microglial cells that were located at 54 µm below zero_0dpi_ during the initial imaging at 0 dpi were found at 12 dpi in 90 µm depth instead. Images obtained at 12 dpi also showed the presence of extravascular parasites inside meninges with higher parasite densities close to the parenchyma border, as previously mentioned. Lateral views of 3D images from infected animals clearly evidenced meningeal space swelling (**Figures 6G, H**-schematic representation). Noteworthy, meningeal space in mock mice was constant. Statistical analysis revealed significant changes between mock and infected mice from 7 dpi onwards (**Figure 6I**), i.e., 2 days after the first rise of parasites in the blood. Thus, the displacement of the field of view in the z-direction is a valuable parameter to appraise meningitis in this sleeping sickness animal model as it is a sensitive and quantifiable parameter.

## 4 Discussion

As microglia are the primary immune cells in the brain parenchyma, it has been assumed that they may take part during the meningo-encephalitic stage of the sleeping sickness (23). Nevertheless, microglia’s involvement in HAT has not been characterized so far, and their study has remained a challenging task (43).

**Figure 7:**
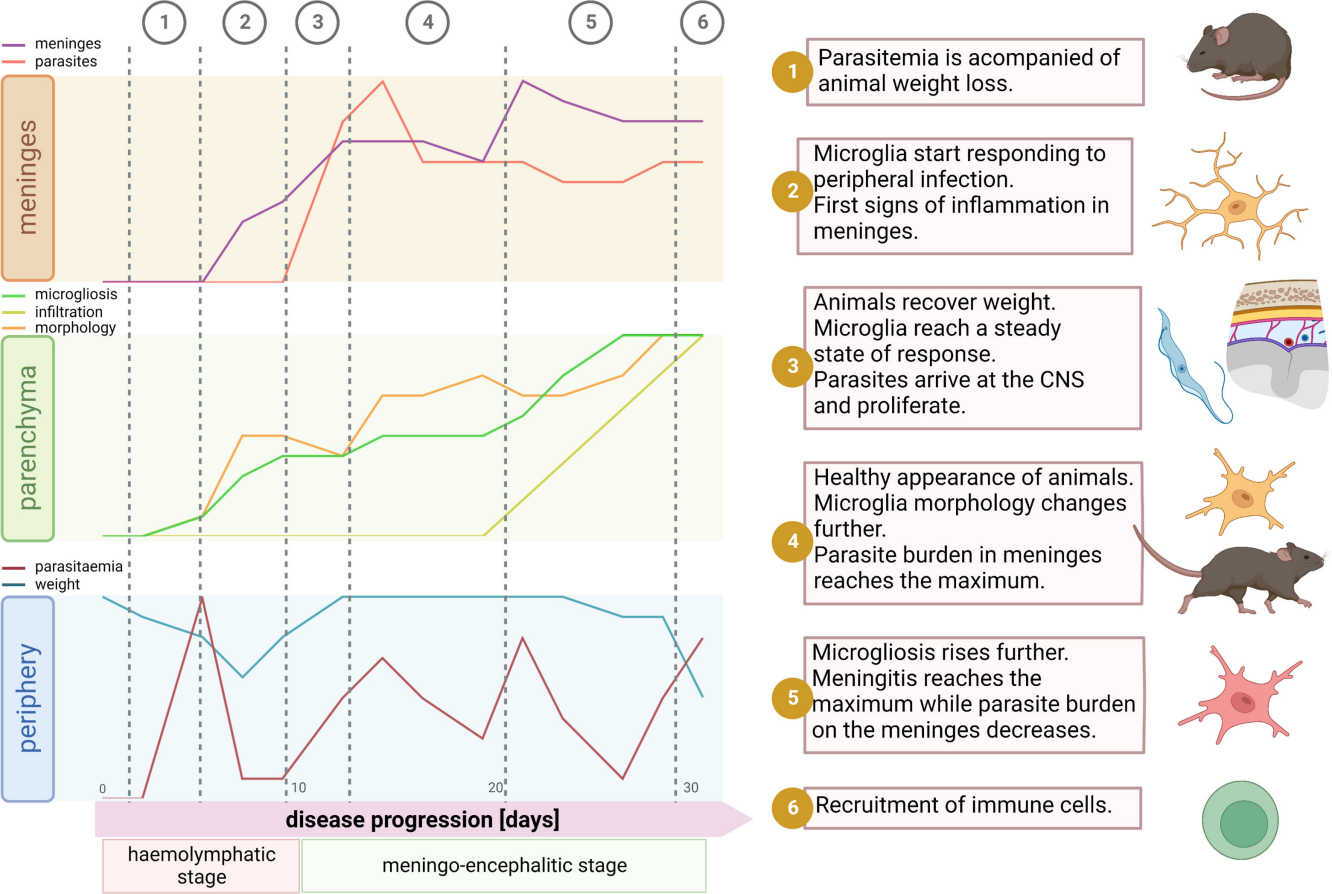
Synopsis of events during disease progression. After parasite inoculation there is an initial phase of local infection without visible symptoms of disease. Around the day 4 parasites start to appear in blood. Typically, there is a peak of parasitemia at day 5, which is accompanied by significant decrease of body weight (1). By this time microglia start responding to the infection. Mainly traits of microglial reaction here comprehend soma volume enlargement, shortening of the total filament length and filament complexity decrease, and an augmented proliferation. These signs of inflammation inside the parenchyma are paralleled in the meninges, where a significant increase of the meningeal thickness appears, however without parasite invasion (2). Then, likely due to decline in parasitemia, the inflammatory response may become weaker; animals recover weight and microglia remain in a steady state without further response (3). Normally by day 12, parasites arrive at the meninges and thus the microglial response restarts devoid of significant delay. After a few days, meningeal parasite burden reaches a ceiling and microglia changes become pronounced and widespread. Although by that time a significant number of immune cells have already infiltrate the meninges, recruitment into the parenchyma is not a palpable feature yet (4). Next, meningeal thickness hits a maximum and meningeal parasite density drops (5). In the final stage of the disease, around day 30, the well-being status of the host declines. Animals experiment weight loss, microglial processes length and complexity plummet, microglia proliferation escalates, and by day 30 a massive infiltration of immune cells into the parenchyma is unmistakable (6). Illustration created using BioRender.

To our knowledge, this is the first study describing detailed morphological spatio-temporal changes of the microglial response to the infection caused by the protozoan parasite *Trypanosoma brucei*. These morphological changes were associated with events underlying disease progression like parasite burden in blood, animal condition, parasite location in the CNS, and meninges inflammation (**Figure 7**). Contrary to belief, we uncovered that *in vivo* cortical microglia start responding to the disease already during the hemolymphatic stage (5 dpi), before the parasite has reached the CNS (typically at 12 dpi). This initial microglial response (evidenced by soma size increase and microglial processes length shortening) might work as a priming phase for later, when the parasite arrives in the CNS, making these immune cells poised to attack intruders efficiently. In addition to cortical microglia, microglia in deeper regions of the brain (like those surrounding the lateral preoptic area, CVOs) also respond early to the infection. In line with these findings, reports have shown that trypanosomes were found in CVOs early upon infection, suggesting that CVOs may serve as a gateway for parasite entry into the CNS (44, 45). Afterward, microglia located around the PVR started displaying changes in number and shape. It is well established that the periphery communicates with the brain by several routes, including the vagal nerve or through cytokines, which in turn reach microglia in CVOs or stimulate BBB’s endothelial cells to produce lipophilic messengers such as prostaglandins that diffuse through the BBB, spreading the signal in the parenchyma (reviewed in refs (46, 47).). Peripheral injection of LPS, known to induce the expression of proinflammatory mediators like TNF-α and IL-1β, causes a microglial response (48, 49). Primary humoral responses against *T. brucei* infection involve the production of proinflammatory molecules such as TNF-α and INF-γ (50–52). Thus, seems reasonable that a peripheral humoral response to trypanosome infection can be sensed by microglia adjacent to the BBB. Therefore, microglial changes around CVOs and perivascular areas appear earlier than those near PVR, which has direct contact with the CSF rather than with the blood.

Moreover, in our experimental model, parasites relocated from the local inoculation site to the bloodstream (4-5 dpi) and subsequently to the CSF (12 dpi). Therefore, anatomical niches where these two fluids communicate with the brain (BBB and pial-CNS barrier) may account for the time-dependent diversity in the microglial response during infection. Indeed, we found that the initial microglial response to the infection (5 dpi) stabilized after a couple of days (~9 dpi) but rebooted right after parasites appeared in the CSF (12 dpi). Remarkably, we found a shift in the maximum microglia response of circa 48 hours after parasitemia reached the highest level but a faster reaction when parasite burden in the CSF increased. It may be due to a priming effect after the first infection stimulus that allowed microglia to respond adeptly to a second stimulus, as it has been seen in an infection model using lipopolysaccharide (LPS) (53).

The response of microglial cells to infection is evident through observable changes in their morphology. These changes were heterogeneous, distinguishing at least three distinct types: proliferative microglia, overgrowing microglia, and no responding microglia. While determining the precise microglial function requires multidimensional integration of transcriptomic, metabolomic, proteomic, epigenetic, and phenotypic (morphology) data (54), some microglial morphology and density alterations can be associated with different roles. The local proliferation of microglia observed in trypanosome-infected mice can lead to the formation of innate immune barriers, which restrict the pathogen spread within the CNS. This is of particular relevance in areas where physical barriers are “weaker” (e.g., nearby CVO). In this scenario, the distance to nearest neighbor (dnn) of microglia will be reduced to ensure a thicker and more efficient network. Indeed, our data show diminished dnn around CVO but also in the cortex. The last may be due to abundance of the BBB in this area (high capillary density with more endothelial cells) that enable enhanced interchange/communication with the periphery (55). In the context of trypanosome-infected mice, microglia tended to increase the volume of their cell bodies and thicken their processes, adopting amoeboid forms commonly associated with phagocytosis. Experimental evidence supports the occurrence of phagocytosis *in vivo* during infection. We observed microglia containing mCherry-labeled material (expressed by trypanosomes) within their cell bodies. Remarkably, trypanosomes directly injected into the parenchyma were unable to survive (56). We previously demonstrated that microglia *in vitro* respond to the presence of the parasite by engulfing and eliminating it through phagocytosis (26). Notably, in the absence of peripheral immune cells infiltrate, microglia are the primary immune cells responsible for phagocytosis in the parenchyma. Microglia containing mCherry were observed from 12 dpi upwards, before the infiltration of inflammatory peripheral cells occurred (after 20 dpi). Furthermore, microglial upregulation of genes associated with antigen presentation were reported in a murine model of chronic human African trypanosomiasis (HAT) (57).

The presence of parasites in the CSF has been extensively demonstrated, and it is a marker for disease staging (58). However, whether this anatomical environment is favorable or not for the parasite’s survival has remained a matter of debate. Earlier studies on the pathogenesis of trypanosome infection showed that, after the first occurrence in the CSF, parasites persist there for weeks until animals die (59). On the contrary, attempts to cultivate trypanosomes *in vitro* in CSF failed, suggesting that the CSF contains trypanotoxic molecules (60). Some neuropeptides were shown to kill trypanosomes *in vitro* (61), although at high concentrations. Interestingly, in our *in vivo* observations, we visualized in all infected mice healthy trypanosomes swimming in the CSF within the subarachnoid space, even conjoined parasites (i.e., parasites undergoing cell division) were detected. It cannot be ruled out that under conditions where the host immune response causes an increase in the concentration of trypanotoxic neuropeptides, the CSF becomes a less permissive environment but is still not fully restrictive. The last assumption is sustained by the fact that the parasite burden in the meninges, despite fluctuations, never disappeared.

In addition to the CSF, parasites were found at the highest density in the CSF-parenchyma border, i.e., directly on the pial cell layer. Microglia located at this proximity showed heightened responses, reflected by increased morphological changes. It might be due to the diffusion of PAMPs through the CSF/ISF system (62) and then into the parenchyma, ultimately sensed by microglia.

During the recordings of the meninges, we detected a significant increase of other immune cells that express CX_3_CR1 in the leptomeninges in the course of the disease progression. A 47-fold increase of T cells inside the meninges at 12 dpi was reported in a similar experimental model as the one used in this study (41). Likewise, in another murine model of HAT a moderate inflammatory cell infiltration into the meninges and the perivascular space of some blood vessels was described (63). We raised the question whether and when inflammatory cells can be recruited into the neuropil. In infected animals, we found a significant immune cell infiltration during the late meningo-encephalitic stage (30 dpi), which was not present or negligible at least up to 20 dpi. These findings are consistent with other studies using mouse models of HAT that showed T cell infiltration after 27-30 dpi (64, 65). In addition, earlier recruitment of T cells was reported in *T. brucei*-infected mice and rats, however, in brain areas not assessed in our study (66, 67). It was demonstrated that the brain has its own system to manage immune responses, i.e., a local immune cell supply chain that bypasses the systemic circulation (68, 69). This CNS-exclusive immune system involves CSF outflow through the glymphatic system to the skull bone marrow and leukocyte migration back toward the meninges (reviewed in ref (70).). This fact may account for the early signs of inflammation detected in the meninges after the first microglial response but before parasite invasion in this compartment. Whether or not the source of the parenchymal immune infiltration is skull bone marrow, systemic circulation, or both and the impact of that on the disease pathology needs to be investigated.

Overall, we conducted long-term *in vivo* phenomics studies of microglia at the single-cell level, monitoring them in intact brains during the progression of sleeping sickness. We achieved this powerful approach by developing an animal model using two-photon microscopy. We were able to observe the individual behavior of microglia and chronologically associate it with the events that occur in real-time in the animal model during the evolution of the entire disease. Altogether, our comprehensive study shows the progressively strong heterogeneous response of microglia during the progression of sleeping sickness and demonstrates an unpredictable early involvement of these cells during the hemolymphatic stage of the disease. This investigation sets the stage for further evaluations to decipher the role of microglia and other immune cells inside the CNS in neurotropic infections before pathogens invade the brain. Combining the long-term *in vivo* model developed here with omics approaches such as epigenomic, proteomic, metabolomic, and transcriptomic will allow researchers to define the state and function of microglia in the progression of neuroinfectious diseases in a spatio-temporal context.

## Data availability statement

The original contributions presented in the study are included in the article/**Supplementary Material**. Further inquiries can be directed to the corresponding author.

## Ethics statement

The animal study was approved by the Regional Council Office (Regierungspräsidum, Tübingen) under the project number PY17/05. The study was conducted in accordance with the local legislation and institutional requirements.

## Author contributions

NU: Formal Analysis, Investigation, Methodology, Supervision, Visualization, Writing – original draft, Writing – review & editing. SG: Formal Analysis, Investigation, Visualization, Writing – review & editing. CR: Formal Analysis, Investigation, Visualization, Writing – review & editing. AS: Investigation, Visualization, Writing – review & editing. EZ: Investigation, Writing – review & editing. MD: Supervision, Writing – review & editing. OG: Conceptualization, Resources, Writing – review & editing. KF: Conceptualization, Data curation, Formal Analysis, Funding acquisition, Investigation, Methodology, Resources, Supervision, Visualization, Writing – original draft, Writing – review & editing.
