# Supplementary material for: Live imaging of microglia during sleeping sickness reveals early and heterogeneous inflammatory responses
Source: Front Immunol. 2023 Sep 13;14:1253648. doi: 10.3389/fimmu.2023.1253648 (PMC10534015; doi:10.3389/fimmu.2023.1253648)
Supplement: Supplementary file 1 [file DataSheet_1.docx]

Supplementary Material

Live imaging of microglia during sleeping sickness reveals early and heterogeneous inflammatory responses

**Nestor L. Uzcategui, Sena Güçer, Cris Richter, Annika Speidel, Elizabeta Zirdum, Michael Duszenko, Olga Garaschuk, Katherine Figarella ^*^**

*** Correspondence:**Katherine Figarella.

Katherine.figarella@uth.tmc.edu

Present address: Anesthesiology, Critical Care and Pain Medicine Department, University of Texas Health Science Center at Houston, 77030 Texas, USA.


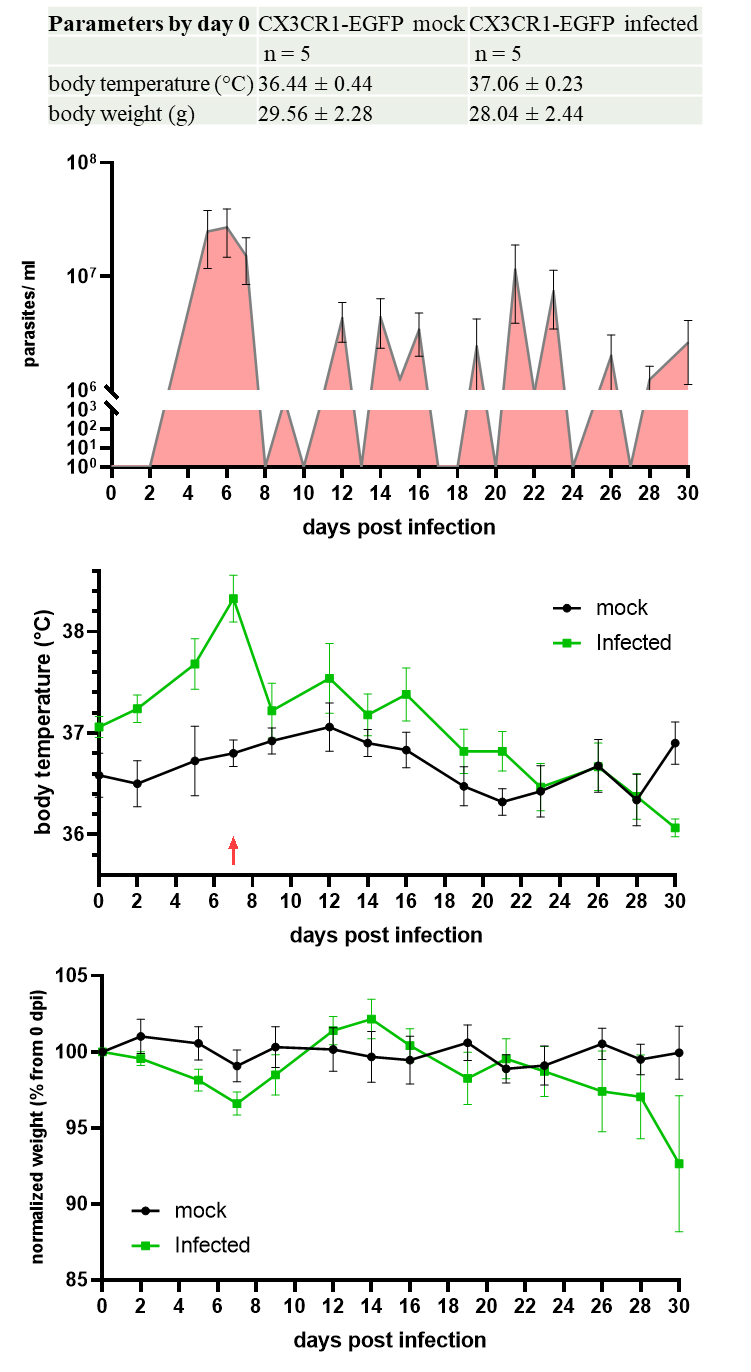
**Supplementary Figure 1.** **Monitoring of *Trypanosoma brucei*-mock and infected** **CX_3_CR1^GFP/+^ transgenic** **mice.** 4-6 months old mice were injected intraperitoneally either with vehicle (mock) or with a parasite suspension (Tbb GVR/35-mCherry) containing 3 × 10^4^ parasites. Basal conditions for body weight and temperature are shown in the inserted table. Parasitemia was monitored in blood taken from the vein tail by direct counting using a hemocytometer. Body temperature was measured using a rectal thermometer. Body weight is represented as the percentage of the starting weight before parasites were inoculated (n=5 mice per condition). Arrow represents statistical significance. Statistical analysis was performed by unpaired t-test and discovery determined using the Two-stage linear step-up procedure of Benjamini, Krieger and Yekutieli, with Q = 1%.


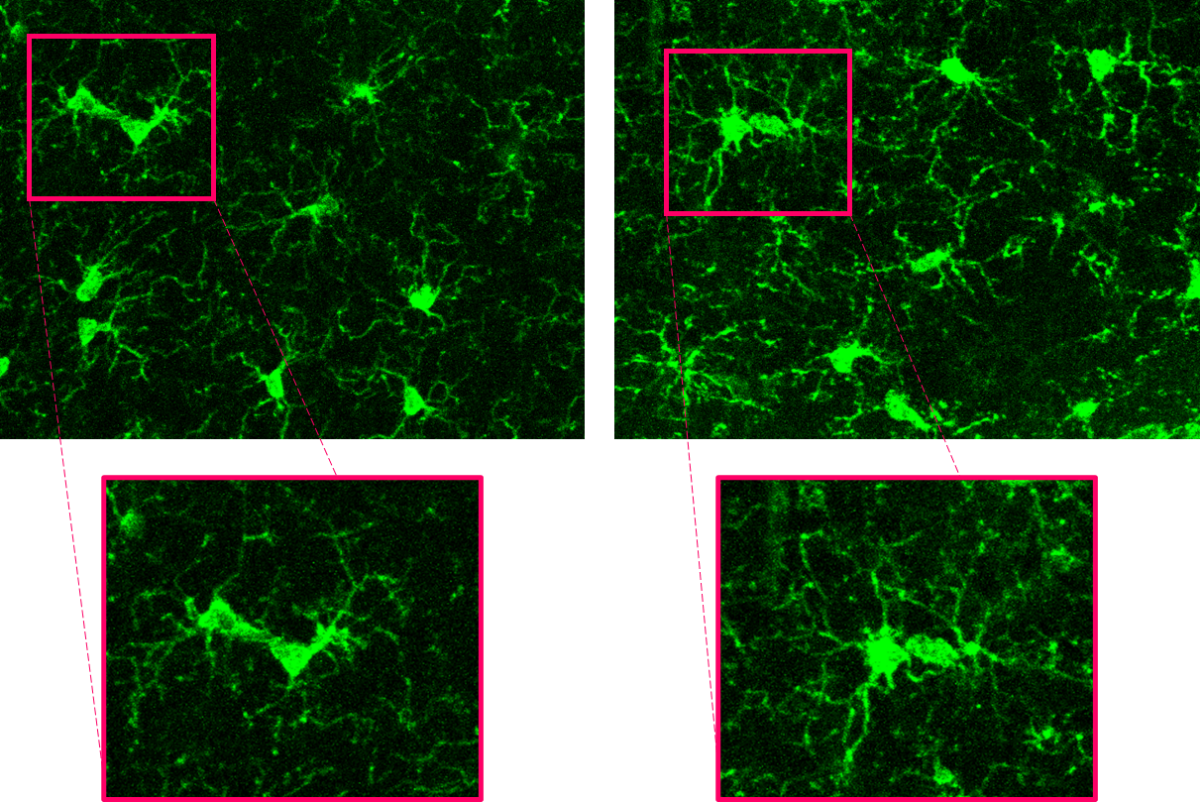


**Supplementary Figure 2.** **Microglia undergoing cell division.** These are representative MIP images of microglia undergoing cell division in trypanosome-infected mice. The images show the dividing microglia and are projected from a depth of 10 µm.

**
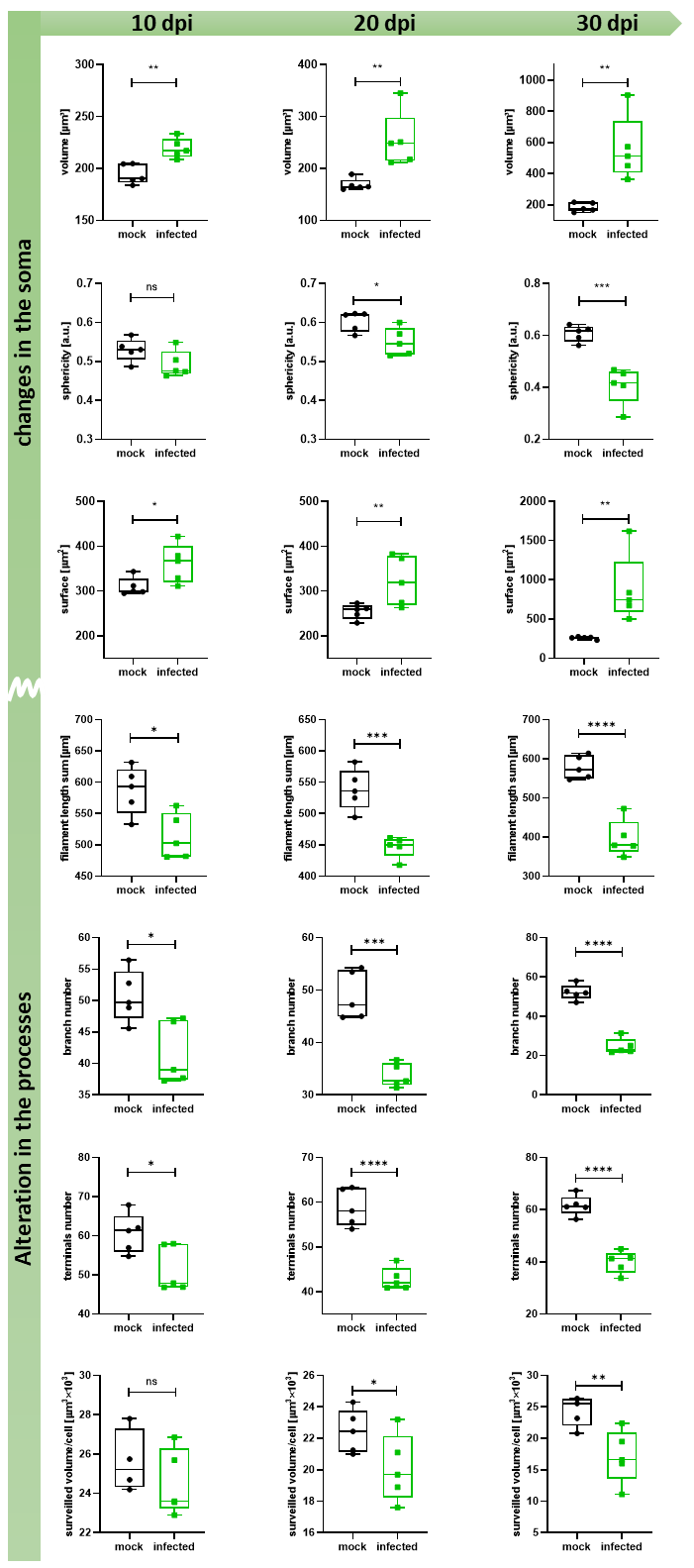
**

**Supplementary Figure 3. Morphometric traits of cortical microglial in mock and infected mice.** Morphological parameters of cortical microglia from mock and infected animals were statistically compared in 10 dpi, 20 dpi, and 30 dpi samples. Whisker graphs show individual values per mouse (n=5 mice per condition) and represent the median with maximum and minimum. Data were analyzed using non-parametric Mann-Whitney test (volume, surface, and number of terminal points) or parametric unpaired t-test with Welch’s correction (sphericity and filament length and branch number). *, p < 0.05; **, p < 0.01; ***, p < 0.001; ****, p < 0.0001 compared with mock mice treated with vehicle.

**Supplementary Figure 4. Microglial soma area from mock and infected CX_3_CR1^GFP/+^ transgenic mice.** Soma area calculations were performed using GECI-Quant in ImageJ as described in the Figure 6. Values are expressed by mouse (n = 5 per condition). Whiskers show individual values per mouse plus the median with minimum and maximum. The mean is marked by +. Normality was analyzed using the Shapiro-Wilk test. Unpaired t-test was used to assess statistical significance. *, p < 0.05; **, p < 0.01.

**Supplementary Video 1. Parasites inside the meninges (z-stack).** Imaging in z-direction of the meningeal space in an infected mouse at 12 dpi. Image was acquired from the upper part of the dura mater till parenchyma border (0-80 µm) to show parasites. Image acquisition was performed at 20x magnification, Zoom3, Kalman filter 2, step 2 µm, and 512 x 512 resolution.

**Supplementary Video 2. Parasites inside the meninges (time series).** Meningeal space in an infected mouse at 14 dpi was imaged at a depth 22 µm below the dura mater to show parasites. Time series acquisition was performed at a frequency of 1.12s/frame (31 frames).

**Supplementary Video 3. Immune cells patrolling inside the meninges.** Area located 15 µm below the dura mater in an infected mouse at 16 dpi. Time series acquisition was performed at a frequency of 1.12 s/frame (49 frames), with an interval of 12.1 s. Total time recorded: 10.08 min.

**Supplementary Video 4. Diapedesis inside the meninges.** Video recording showing extravasation of cells into the meningeal space in an infected mouse (21 dpi). Area located 48 µm below the dura mater. Time series acquisition was performed at a frequency of 1.12s/frame (60 frames).

**Supplementary Table 1.** Score sheet to evaluate animal well-being during the disease progression

| **Parameter** | **Observation** | **Score^*^** |
| --- | --- | --- |
| body weight | unaffected/increase | 0 |
|  | reduction up to 10% | 1 |
|  | reduction 11-15% | 2 |
|  | reduction >15 and <20% | 4 |
|  | reduction from 20% | 6 |
| Breathing | regular | 0 |
|  | irregular | 3 |
|  | difficult | 5 |
| Vigilance | normal/awake | 0 |
|  | strongly aroused | 1 |
|  | dull/sluggish ≤ 24h | 1 |
|  | somnolence | 2 |
|  | Sopor | 3 |
|  | Apathy | 4 |
| Mobility | spontaneous/unrestricted | 0 |
|  | slowed down/powerless ≤ 24h | 2 |
|  | slowed down/powerless > 24h | 3 |
|  | spontaneous/fluctuating | 3 |
|  | reduced mobility provoked by manual touch | 5 |
| General condition | Pelage: smooth, shiny / Eyes: open, clear, shiny / Orifices: clean | 0 |
|  | Pelage: shaggy, piloerection / Eyes: narrowed, cloudy / Orifices: unkempt body orifices ≤ 24h | 2 |
|  | Pelage: shaggy, piloerection / Eyes: narrowed, cloudy / Orifices: unkempt body orifices > 24h | 3 |
|  | Pelage: bristling, bucking / Eyes: narrowed to closed, cloudy / Orifices: sticky, moist, with or without central nervous disorders (tremors, spasms, paralysis) | 5 |
|  | **Additional Criteria** |  |
| Wound edges | closed, dry, non-irritable | 0 |
|  | reddened, wound exudate | 2 |
|  | swollen, increased exudation | 5 |
|  | severely swollen, heavy exudation, primarily pus | 5 |
| Body temperature (during infection) | 36-37°C | 0 |
|  | deviation by ≤ 1°C | 1 |
|  | deviation by >1 - 2°C | 2 |
|  | deviation > 2°C | 5 |

(*): Score 0: normal.

Score 1-2: low stress. Requires observation at least once every 24 hours.

Score 3-5 (<12h): moderate stress. Requires observation at least once every 12 hours.

Score 4-5 (>24h): high stress. Demands consultation with veterinarians.

Score ≥ 6: Stipulates discontinuation of the experiment and immediate euthanasia.
